# Combination of *Aspergillus niger* culture and glycyrrhizic acid alleviates the toxic effects of multi-mycotoxins on broiler production performance and nutrient metabolism

**DOI:** 10.3389/fnut.2025.1628442

**Published:** 2025-07-25

**Authors:** Jinqiu Tu, Mengke Li, Ping Wang, Lijun Wang, Sanjun Jin, Xinxin Li, Juan Chang, Qingqiang Yin, Chaoqi Liu, Qun Zhu, Maolong Li, Fushan Lu

**Affiliations:** ^1^College of Animal Science and Technology, Henan Agricultural University, Zhengzhou, China; ^2^Henan Delin Biological Product Co. Ltd., Xinxiang, China; ^3^Henan Puai Feed Co. Ltd., Zhoukou, China

**Keywords:** multi-mycotoxin, detoxification, broiler, growth performance, serum metabolomics

## Abstract

**Introduction:**

Mycotoxins in animal diets cause a lot of economic loss in animal husbandry annually. The objective of this experiment was to evaluate the effect of combination of *Aspergillus niger* culture and glycyrrhizic acid (CANCGA) on alleviating multi-mycotoxin toxicity for broiler production performance and nutrient metabolism.

**Methods:**

A total of 500 one-day-old male broilers were randomly divided into 10 groups, 5 replications in each group and 10 broilers in each replication. The feeding period was 21 d. The dietary treatment included group A (the basal diet as the control group); group B (0.03 mg/kg aflatoxin B_1_ (AFB_1_) + 0.15 mg/kg zearalenone (ZEN) + 1.5 mg/kg deoxynivalenol (DON), low-dose mycotoxin diet); group C (0.07 mg/kg AFB_1_ + 0.5 mg/kg ZEN + 3.0 mg/kg DON, high-dose mycotoxin diet); groups D, E and F (basal diet supplemented with 0.2, 0.4 and 0.6 g/kg CANCGA, respectively); groups G, H and I (low-dose mycotoxin diet supplemented with 0.2, 0.4 and 0.6 g/kg CANCGA, respectively); group J (high-dose mycotoxin diet supplemented with 0.4 g/kg CANCGA).

**Results:**

The results demonstrated that broiler mortality in groups B and C was 2 and 6%, which in other groups was zero, indicating that CANCGA addition in diets could decrease broiler mortality caused by multi-mycotoxins. Average daily weight (ADG), metabolic rates of protein and phosphorus were significantly declined, while the ratio of daily feed intake and daily gain were significantly increased when dietary mycotoxin concentration was increased (*p* < 0.05). Compared with the control group, low-dose mycotoxin in diet could increase serum alanine aminotransferase (ALT) and aspartate aminotransferase (AST) activity (*p* < 0.05), while decrease serum total protein (TP), albumin (ALB) and total cholesterol (TC) levels (*p* < 0.05). However, CANCGA addition could effectively reverse the above results. Compared with the low-dose mycotoxin group, the addition of 0.4 g/kg CANCGA could decrease serum ALT, AST, alkaline phosphatase (ALP), glucose (GLU), triglyceride (TG) and high-density lipoprotein (HDL) levels (*p* < 0.05), while increase ALB, TC levels and ALB/Globulin (GLB) (*p* < 0.05), indicating that CANCGA addition was able to reduce oxidative stress of broilers induced by multi-mycotoxins. The contents of residual AFB_1_, ZEN and DON in broiler excreta were significantly increased in the low-dose mycotoxin group (*p* < 0.05), compared to the control group; however, CANCGA addition could decrease AFB_1_, ZEA and DON contents in broiler excreta. Serum metabolomics showed that metabolites such as creatine, N-acetyl-L-phenylalanine and linoleic acid as well as metabolic pathways related to glycine, serine, threonine, cysteine, methionine, selenium compounds and linoleic acid metabolisms were regulated by CANCGA addition to alleviate nutrient metabolic disorders caused by multi-mycotoxins.

**Discussion:**

In conclusion, CANCGA was found to be effective in alleviating multi-mycotoxin toxicity for broilers’ growth performance through reducing oxidative stress and positively regulating nutrient metabolisms.

## 1 Introduction

Mycotoxins were toxic metabolites produced by *Aspergillus* spp., *Penicillium* spp., *Fusarium* spp. and other fungi during their growth process (1). Over 300 kinds of mycotoxins were identified that pose harm to both humans and animals (2). Among them, the most potent and wide-distribution mycotoxins included aflatoxin B_1_ (AFB_1_), zearalenone (ZEN) and deoxynivalenol (DON) (3). These mycotoxins could be generated at many stages including crop cultivation, processing, transportation and storage of grains and their by-products due to variations in environmental ask temperature and humidity (4, 5), posing significant health risks to both humans and animals. In addition, the main component of an animals’ diet consists mainly of grains, which are a favorable substrate for mycotoxin-producing fungal species. These ingredients have high inclusion rates in animal compound feed, and if contaminated, could be a source of contamination of the final products (6, 7). Up to 88% of 74,821 samples of diets and feed ingredients (e.g., corn, wheat and soybeans) collected from 100 countries were polluted with multi-mycotoxins, in which AFB_1_, ZEN and DON were detected in 23, 64 and 45% of the samples, respectively (8). It was reported that 17,316 samples of feed and feed raw materials from all over the world were analyzed for contamination with aflatoxins, ochratoxin A, zearalenone, deoxynivalenol and fumonisins, in which 72% of the samples were tested to be positive for at least one mycotoxin and 38% were found to be co-contaminated (9).

The presences of mycotoxins are associated with carcinogenicity, teratogenicity, hepatotoxicity, nephrotoxicity, embryotoxicity and immunosuppression in animals (10–13). Moreover, their occurrence could reduce body weight and feed conversion rates, increase incidences of diarrhea and mortality in poultry (14, 15). AFB_1_ was demonstrated to significantly disrupt hepatic lipid and protein metabolism in animals, which can lead to liver function damage and affect production performance (16–18). ZEN, an estrogen analog, competed with endogenous estrogen for receptor binding sites upon entering the organism, results in reproductive toxicity (19–21). DON contamination in animal feeds causes impairment of intestinal barrier function (22, 23), leading to intestinal flora disorders in animals (24). Once DON is absorbed into the body, it inhibits protein synthesis (1, 25, 26). However, mycotoxin contamination in feedstuffs or diets typically involves multiple toxin types rather than single toxin. It was observed that low concentration of multiple mycotoxins has a greater detrimental impact on livestock than high concentration of single mycotoxin (27–29). Coexistence of AFB_1_, ZEN and DON in poultry diets could lead to an exacerbation of health issues in animals and result in diminished economic returns due to their combined toxicity (30). In addition, feeds contaminated with AFB_1_, ZEN and DON resulted in disruption of amino acid metabolic pathways such as alanine, aspartic acid and glutamine (31) as well as impacting blood glucose metabolites such as glycine, arginine and tryptophan (32).

Therefore, effectively addressing the risks associated with multi-mycotoxins has become an urgent priority. Currently, three primary approaches were employed to mitigate or eliminate mycotoxin risks: physical, chemical, and biological methods (33). Among these options, biological detoxification methods were considered specific, efficient, and environmentally friendly. For instance, the previous report showed that fourteen strains of *Aspergillus niger* isolated from peanuts demonstrated complete inhibition of AFB_1_ production through co-culturing (34). It was discovered that one strain of *Aspergillus niger* consistently degraded ZEN by over 95%, resulting in the formation of low-toxicity products (35). Glycyrrhizic acid (GA), the main active compound extracted from *Glycyrrhiza glabra*, has been shown to alleviate inflammation, oxidative stress, and apoptosis (36). Also, GA is considered an effective treatment for liver diseases (37). Its combination with probiotic complexes attenuated DON-induced oxidative stress, inflammation and apoptosis in IPEC-J2 cells (38). However, the combined effect of GA and *Aspergillus niger* in reducing AFB_1_ induced toxicity has not been studied. After considering both functions of GA and *Aspergillus niger*, the combination of *Aspergillus niger* culture and GA (CANCGA) was used in this study for alleviating multi-mycotoxin toxicity caused by AFB_1_, ZEN and DON in broiler production, so as to assess their mitigation potential and provide a foundation for addressing issues related to multiple mycotoxin contamination.

## 2 Materials and methods

The study and included experimental procedures were approved by the guidelines of Animal Care and Use Ethics Committee of Henan Agricultural University (SKLAB-B-2010-003-01). All animal experiments were conducted in strict accordance with the institutional guidelines for care and use of laboratory animals. Animal feeding experiment was conducted in a chicken farm of Henan Agricultural University.

### 2.1 CANCGA and mycotoxin preparation

*Aspergillus niger* with degrading AFB_1_ and ZEN was preserved in the Laboratory of Animal Nutrition and Feed Biotechnology in Henan Agricultural University. GA was provided by Henan Delin Biological Products Co., Ltd. The preparation of solid culture of Aspergillus niger was made by mixing the three feed ingredients of bran, soybean meal and corn in the ratio of 7:2:1, taking 10 g of the mixture in a triangular flask, after sterilization, adding 1 mL of *Aspergillus niger* seed solution and 5 mL of sterile water, mixing thoroughly and cultivating at 30°C for 5–7 d and then taking it out and drying and crushing the mixture (39). The mycotoxin degradation experiments *in vitro* confirmed that the degradation rates of AFB_1_ and ZEA were 60.40 and 97.67%, respectively, when 0.04% *Aspergillus niger* culture was applied. The further research indicated that the degradation rate of AFB_1_ was increased to 68, 71 and 63% when 0.02, 0.04 and 0.06% of GA were added. In addition, previous study in our laboratory confirmed that GA could alleviate the damage of intestinal cells caused by DON (38). Therefore, CANCGA was prepared by combining *Aspergillus niger* culture with GA at both 0.04% addition (mixed in ratio 1:1 ratio) for alleviating toxicity of AFB_1_, ZEN and DON in the further broiler feeding experiment.

### 2.2 Diet preparation and animal management

500 one-day-old male Arbor Acres (AA) broilers were divided into 10 groups, each group consisting of 5 replications with 10 broilers per replication. The broilers were reared in cages, allowing free access to diet and water. Room temperature was around 25°C, but the temperature under nurturing umbrella was 33–35°C, 29–32°C and 26–28°C for one-week-old, two-week-old and three-week-old broilers, respectively. The relative humidity was kept at 60 to 65%. The experimental period was 21 d. Daily feed intake, and dead broilers were recorded daily. Broiler body weight in each replication was weighted at the age of 1 and 22 d. Parameters such as average daily gain (ADG), average daily feed intake (ADFI), feed-to-gain ratio (F/G) and mortality were calculated. Additionally, a standard immunization program was implemented within the first one week. The experimental groups were organized as follows:

Group A: the basal diet as the control group (0.002 mg/kg AFB_1_ + 0.041 mg/kg ZEN + 0.946 mg/kg DON).
Group B: low-dose mycotoxin diet (0.03 mg/kg AFB_1_ + 0.15 mg/kg ZEN + 1.5 mg/kg DON).
Group C: high-dose mycotoxin diet (0.07 mg/kg AFB_1_ + 0.5 mg/kg ZEN + 3.0 mg/kg DON).
Group D: basal diet supplemented with 0.2 g/kg CANCGA.
Group E: basal diet supplemented with 0.4 g/kg CANCGA.
Group F: basal diet supplemented with 0.6 g/kg CANCGA.
Group G: low-dose mycotoxin diet supplemented with 0.2 g/kg CANCGA.
Group H: low-dose mycotoxin diet supplemented with 0.4 g/kg CANCGA.
Group I: low-dose mycotoxin diet supplemented with 0.6 g/kg CANCGA.
Group J: high-dose mycotoxin diet supplemented with 0.4 g/kg CANCGA.

The basal diets were formulated based on the broiler feeding standards outlined in the NRC (1994) guideline. To adjust AFB_1_ and ZEN contents in diets, normal corn in the basal diet was substituted with moldy corn, while DON content was adjusted with corn by-product. The diet formulation and nutrient levels were listed in Table 1.

**Table 1:** Compositions and nutrient levels in broiler diet (%, air-dried base).

Compositions	Basic diet	Low-dose mycotoxin	High-dose mycotoxin
Corn	56.82	44.00	29.05
Mold corn meal	0	12.00	24.00
Soybean meal	34.90	28.57	15.70
Corn by-products	0	7.00	22.00
Fish meal	1.80	1.80	1.80
Soybean oil	3.20	3.35	4.00
CaHPO_4_	1.15	0.42	0.00
CaCO_3_	1.30	1.88	2.16
Methionine	0.23	0.23	0.23
Lysine	0.00	0.15	0.46
Salt	0.30	0.30	0.30
Premix^a^	0.30	0.30	0.30
Total	100.00	100.00	100.00
Nutritional levels^b^			
ME (MJ/kg)	12.58	12.55	12.53
CP	23.16	23.93	23.80
Ca	1.08	1.17	1.21
Total P	1.28	1.21	1.01
Available P	0.30	0.30	0.30
Lysine	1.16	1.15	1.15
Methionine	0.57	0.57	0.57

CP = crude protein; DE = digestible energy; Ca = calcium; P = phosphorus; NDF = neutral detergent fiber; ADF = acid detergent fiber.

a Premix provides (per kg diet): VA 12000 IU; VD_3_ 3,000 IU; VE 20 IU; VK_3_ 1.0 mg; VB_1_ 2.0 mg; Riboflavin (VB_2_) 6 mg; Nicotinic acid (niacin) 35 mg; Choline 1.3 g; Calcium pantothenate 10 mg; VB_6_ 3.5 mg; VB_12_ 0.01 mg; Biotin 0.15 mg; Folic acid 1.25 mg; Copper (copper sulfate) 8 mg; Iron (ferrous sulfate) 100 mg; Manganese (manganese sulfate) 80 mg; Zinc (zinc oxide) 60 mg; Iodine I (calcium iodate) 0.45 mg; Selenium (sodium selenite) 0.35 mg. The crude protein, calcium and total phosphorus levels are measured, and the others are calculated.

b The CP, DE, Ca, P, cellulose and hemicellulose levels were measured, whereas the others were calculated.

### 2.3 Sample collection and treatment

Excreta collection of broilers in each replication was performed on days 18–20 of the feeding experiment, followed by spraying with 10% sulfuric acid solution for nitrogen fixation, dried at 65°C, and ground for further use. At day 21, blood samples (2 mL) were collected from the wing vein of five broilers in each group and stored in a refrigerator at 4°C until serum precipitation occurred in the collection tube. Subsequently, centrifugation was conducted at 1,520 × *g* for 10 min to obtain serum, which was then transferred to a sterilized tube and stored at −80°C for future utilization.

### 2.4 Determinations of nutrient metabolic rates and residues of AFB_1_, ZEN, and DON

The crude protein (CP), ether extract (EE), calcium (Ca) and phosphorus (P) contents in diets and excreta were determined according to the methods of national standards GB/T 6432–2018, GB/T 6433–2006, GB/T 6436–2002 and GB/T 6437–2018, respectively. The calculation of nutrient metabolic rates was as follows: Nutrient metabolic rate (%) = 100 × (nutrient content in diet−nutrient content in excreta)/nutrient content in diet. Based on the growth performance of broilers in each group at 21 d, broilers in groups A, B, E and H were selected for the determination of excreta toxin residues. The contents of AFB_1_, ZEN and DON in excreta were detected according to the protocol of Suwei toxin detection kits (Suwei Biological Research Co., Ltd. Jiangsu, China). The calculation of AFB_1_ degradation rate was as follows: AFB_1_ degradation rate (%) = (AFB_1_ content in control group−AFB_1_ content in test group)/AFB_1_ content in control group × 100. DON and ZEN degradation rates were calculated in the same way as AFB_1_.

### 2.5 Determination of serum biochemical parameters

Using a fully-automated blood biochemistry analyzer to measure the serum contents of glucose (GLU), triglyceride (TG), high-density lipoprotein (HDL), total cholesterol (TC), low-density lipoprotein (LDL), aspartate aminotransferase (AST), lactate dehydrogenase (LDH), alkaline phosphatase (ALP), alanine transaminase (ALT), total protein (TP) and albumin (ALB).

### 2.6 Serum pretreatment

Based on the growth performance of broilers in each group at 21 d, broilers in groups A, B, E and H were selected for serum metabolomics analysis. Serum sample was thawed at 4°C and vortexed for 1 min using a vortex mixer (BE-2600, Haimen Qilin Bell Instrument Manufacturing Co., Ltd., Nantong, China) to ensure thorough mixing. And then transferred into 2 mL centrifuge tube. Subsequently, 400 μL methanol (stored at −20°C) was added and vortexed for 1 min prior to centrifugation. The sample was centrifuged at 13,680 × *g* and 4°C for 10 min using a refrigerated centrifuge (H1850-R, Hunan Xiangyi Laboratory Instrument Development Co., Ltd., Changsha, China). The supernatant was carefully transferred to another 2 mL centrifuge tube, then concentrated and dried before being dissolved in 150 μL 4 mg/L 2-chloro-L-phenylalanine (prepared with 80% methanol). The resulting supernatant was filtered through a 0.22 μm filter membrane before being added to the UPLC-MS vial for UPLC-MS detection.

### 2.7 Analysis of UPLC-MS

MS/MS analysis was performed using the Thermo Vanquish Ultra High-Performance Liquid Chromatography (UHPLC) system (Vanquish, Thermo, Massachusetts, United States) coupled with an ACQUITY UPLC^®^ HSS T3 column (2.1 × 100 mm, 1.8 μm). In positive ion mode, the mobile phases consisted of 0.1% formic acid diluted in water (A1) and 0.1% formic acid diluted in acetonitrile (B1); in negative ion mode, the mobile phases were 5 mM ammonium formate diluted in water (A2) and acetonitrile (B2). Thermo Orbitrap Exploris 120 mass spectrometer (Orbitrap Exploris 120, Thermo, Massachusetts, United States) was used to determine the serum metabolites. Mass spectrometry parameters were as follows: positive ion spray voltage of 3.50 kV, negative ion spray voltage of −2.50 kV.

### 2.8 Statistical analyses

The animal experimental data were expressed as means ± standard error. The one-way ANOVA test was conducted using SPSS 26.0. Statistical significance was considered at *p* < 0.05.

The data from metabolomics were processed by XCMS in R package for peak detection, filtering, and alignment of raw mass spectrometry files converted by Proteowizard. Substance identification was conducted by searching and comparison with spectral databases such as HMDB, MassBank, LipidMaps, mzCloud, KEGG, and metabolite standards database of Nomi Metabolism. Differential metabolites were selected based on criteria of *p* < 0.05 and VIP > 1. Spearman correlation analysis was performed between differential metabolites and production performance, serum biochemistry or three kinds of toxin residues in excreta by using Nomi Metabolism platform.

Multivariate statistical methods including downscaling and categorization such as principal component analysis (PCA) and orthogonal-partial least squares discriminant analysis (OPLS-DA) were employed to analyze the data from serum metabolomics in order to identify the differential metabolites between different groups. The significant differences were determined by t-test and the variable importance projection (VIP) of the first principal component of OPLS-DA. A KEGG pathway enrichment analysis was conducted for the identified differential metabolites to evaluate their potential roles in biological responses.

## 3 Results

### 3.1 Effects of CANCGA addition on broiler growth performance

As presented in Table 2, mortality was significantly increased in the low-dose or high-dose mycotoxin groups, compared to the control group. However, supplementation with CANCGA made mortality become zero to remain consistent with the control group, indicating that CANCGA was able to alleviate multi-mycotoxin toxicity for broilers. Moreover, compared to the basal diet, a significant (*p* < 0.05) improvement of ADG and final body weight were observed when 0.4 g/kg CANCGA was added to the basal diet, while final body weight, ADG, ADFI, and F/G were not significantly different when 0.2 g/kg and 0.6 g/kg CANCGA was added to the basal diet. Notably, final body weight and ADG were significantly higher with the addition of 0.4 g/kg CANCGA to the basal diet than with the 0.2 g/kg addition (*p* < 0.05). ADG and ADFI were significantly decreased, while F/G was significantly increased for broilers subjected to high-dose mycotoxin diet, compared to both basal and low-dose mycotoxin diets (*p* < 0.05).

**Table 2:** Effects of CANCGA on growth performance of broilers (*n* = 5).

Groups	Initial body weight, g	Final body weight, g	ADG^11^, g	ADFI^12^, g	F/G^13^	Mortality, %
A^1^	44.10 ± 0.22	762.50 ± 15.03^bc^	34.20 ± 0.71^bc^	51.65 ± 1.68^a^	1.51 ± 0.03^c^	0
B^2^	44.20 ± 0.27	775.08 ± 41.38^abc^	34.80 ± 1.98^abc^	52.67 ± 2.68^a^	1.52 ± 0.13^c^	2
C^3^	44.76 ± 0.34	363.67 ± 11.85^d^	15.19 ± 0.56^d^	40.52 ± 2.65^b^	2.67 ± 0.18^b^	6
D^4^	45.00 ± 0.00	755.24 ± 20.01^c^	33.81 ± 0.95^c^	49.44 ± 3.31^a^	1.46 ± 0.11^c^	0
E^5^	44.50 ± 0.61	808.20 ± 35.16^a^	36.37 ± 1.68^a^	53.36 ± 3.38^a^	1.47 ± 0.03^c^	0
F^6^	44.60 ± 0.42	791.83 ± 19.68^ab^	35.58 ± 0.95^ab^	52.79 ± 2.20^a^	1.48 ± 0.04^c^	0
G^7^	44.23 ± 0.35	751.52 ± 19.38^c^	33.73 ± 0.93^c^	51.29 ± 1.48^a^	1.52 ± 0.06^c^	0
H^8^	45.03 ± 0.13	778.08 ± 16.78^abc^	34.91 ± 0.80^abc^	52.63 ± 0.82^a^	1.51 ± 0.06^c^	0
I^9^	44.84 ± 0.53	775.27 ± 19.17^abc^	34.78 ± 0.92^abc^	51.09 ± 2.24^a^	1.47 ± 0.05^c^	0
J^10^	44.30 ± 0.27	350.83 ± 35.46 ^d^	14.60 ± 1.69^d^	42.31 ± 6.93 ^b^	2.90 ± 0.55 ^a^	0

1 A: Basic diet.

2 B: Low-dose mycotoxin group.

3 C: High mycotoxin group.

4 D: Basic diet + 0.2 g/kg CANCGA.

5 E: Basic diet + 0.4 g/kg CANCGA.

6 F: Basic diet + 0.6 g/kg CANCGA.

7 G: Low-dose mycotoxin group + 0.2 g/kg CANCGA.

8 H: Low-dose mycotoxin group + 0.4 g/kg CANCGA.

9 I: Low-dose mycotoxin group + 0.6 g/kg CANCGA.

10 J: High-dose mycotoxin group + 0.4 g/kg CANCGA.

11 ADG: Average daily gain.

12 ADFI: Average daily feed intake.

13 Feed to gain ratio.

^a–d^ The different lowercase letters in the same column indicate significant differences (*p* < 0.05), while the same lowercase letters in the same column indicate insignificant differences (*p* > 0.05).

### 3.2 Effects of CANCGA on nutrient metabolic rates for broilers

Table 3 showed that high-dose mycotoxin diets without or with CANCGA addition significantly decreased CP and P metabolic rates, while increased EE metabolic rate, compared with the control group (*p* < 0.05). CANCGA addition in high-dose mycotoxin diet had insignificant effect on nutrient metabolic rates. P metabolic rate was significantly decreased (*p* < 0.05), while EE metabolic rate was significantly increased with dietary mycotoxin levels increasing (*p* < 0.05). Compared with the control group, low-dose mycotoxin diet had insignificant effect on other nutrient metabolic rates in spite of increasing EE metabolic rate (*p* < 0.05). In low-dose mycotoxin diets, 0.4 g/kg CANCGA addition significantly increased EE metabolic rate, 0.6 g/kg CANCGA addition significantly decreased Ca metabolic rate, compared with the low-dose mycotoxin diet without CANCGA addition (*p* < 0.05). Compared with the control group, 0.4 g/kg CANCGA addition in the basal diet significantly decreased EE and Ca metabolic rates (*p* < 0.05), while 0.2 g/kg and 0.6 g/kg CANCGA additions in the basal diet had insignificant effect on nutrient metabolic rates. In addition, CANCGA added at 0.4 g/kg in the low mycotoxin group had significantly higher metabolic rates of EE and P than the 0.2 g/kg addition (*p* < 0.05).

**Table 3:** Effects of CANCGA on nutrient metabolic rates for broilers (%, *n* = 5).

Groups	CP^11^	EE^12^	Ca^13^	P^14^
A^1^	65.17 ± 3.96^ab^	68.32 ± 2.55^c^	57.23 ± 5.07^ab^	66.50 ± 3.03^ab^
B^2^	64.02 ± 2.00^abc^	75.35 ± 0.64^b^	55.85 ± 3.26^abc^	62.70 ± 0.98^bcd^
C^3^	58.79 ± 1.78^cd^	83.38 ± 2.15^a^	53.62 ± 3.71^bcd^	43.59 ± 5.50^e^
D^4^	66.05 ± 0.30^ab^	65.97 ± 3.07^cd^	55.30 ± 3.96^abc^	62.45 ± 3.05^bcd^
E^5^	70.17 ± 5.96^a^	62.80 ± 1.26^d^	49.44 ± 0.53^cd^	69.40 ± 6.38^a^
F^6^	67.61 ± 1.00^ab^	67.28 ± 3.16^cd^	52.97 ± 2.24^bcd^	63.83 ± 2.31^abc^
G^7^	62.02 ± 3.48^bc^	75.49 ± 5.06^b^	55.64 ± 3.35^abc^	56.96 ± 5.23^d^
H^8^	68.20 ± 1.73^ab^	82.37 ± 1.92^a^	61.11 ± 2.92^a^	64.54 ± 1.78^abc^
I^9^	67.32 ± 3.83^ab^	78.66 ± 4.65^ab^	48.70 ± 3.20^d^	58.66 ± 5.24^cd^
J^10^	55.04 ± 3.43 ^d^	78.98 ± 5.61 ^ab^	55.70 ± 4.67 ^abc^	41.58 ± 2.14 ^e^

1 A: basic diet.

2 B: low-dose mycotoxin group.

3 C: high mycotoxin group.

4 D: basic diet + 0.2 g/kg CANCGA.

5 E: basic diet + 0.4 g/kg CANCGA.

6 F: basic diet + 0.6 g/kg CANCGA.

7 G: low-dose mycotoxin group + 0.2 g/kg CANCGA.

8 H: low-dose mycotoxin group + 0.4 g/kg CANCGA.

9 I: low-dose mycotoxin group + 0.6 g/kg CANCGA.

10 J: high-dose mycotoxin group + 0.4 g/kg CANCGA.

11 CP: crude protein.

12 EE: ether extract.

13 Ca: calcium.

14 P: phosphorus.

^a–d^ The different lowercase letters in the same column indicate significant differences (*p* < 0.05), while the same lowercase letters in the same column indicate insignificant differences (*p* > 0.05).

### 3.3 Effects of CANCGA on mycotoxin contents in excreta of broilers

Based on the feeding experiment, the excreta and serum samples from groups A, B, E and H were selected as the representative ones for further analyses. As shown in Table 4, the contents of residual AFB_1_, ZEN and DON in broiler excreta were significantly increased in the low-dose mycotoxin group, compared to the control group (*p* < 0.05). Furthermore, compared to the basal diet, residual AFB_1_ content was significantly decreased when 0.4 g/kg CANCGA was added in the basal diet (*p* < 0.05); however, CANCGA addition had the tendency to decrease ZEN and ZON contents in excreta. Compared to the low-dose mycotoxin group, ZEN residue was significantly decreased by 0.4 g/kg CANCGA addition (*p* < 0.05); however, CANCGA addition had the tendency to decrease AFB_1_ and ZON contents in excreta.

**Table 4:** Effects of CANCGA on toxin contents in the excreta of broilers (μg/kg, *n* = 5).

Group	AFB_1_	ZEN	DON
A^1^	5.70 ± 0.54^b^	2.42 ± 0.25^c^	190.69 ± 4.16^b^
B^2^	8.64 ± 0.72^a^	7.37 ± 0.59^a^	222.68 ± 13.74^a^
E^3^	3.30 ± 0.24^c^	1.86 ± 0.28^c^	187.35 ± 12.67^b^
H^4^	8.52 ± 0.85^a^	6.21 ± 0.47^b^	216.65 ± 10.60^a^

1 A: basal diet.

2 B: low-dose mycotoxin group.

3 E: basal diet + 0.4 g/kg CANCGA.

4 H: low-dose mycotoxin group + 0.4 g/kg CANCGA.

^a–c^ The different lowercase letters in the same row indicate significant differences (*p* < 0.05), whereas the same lowercase letters or without lowercase letters in the same row indicate insignificant differences (*p* > 0.05).

### 3.4 Effect of CANCGA on serum biochemical parameters of broilers

**Figure 1:**
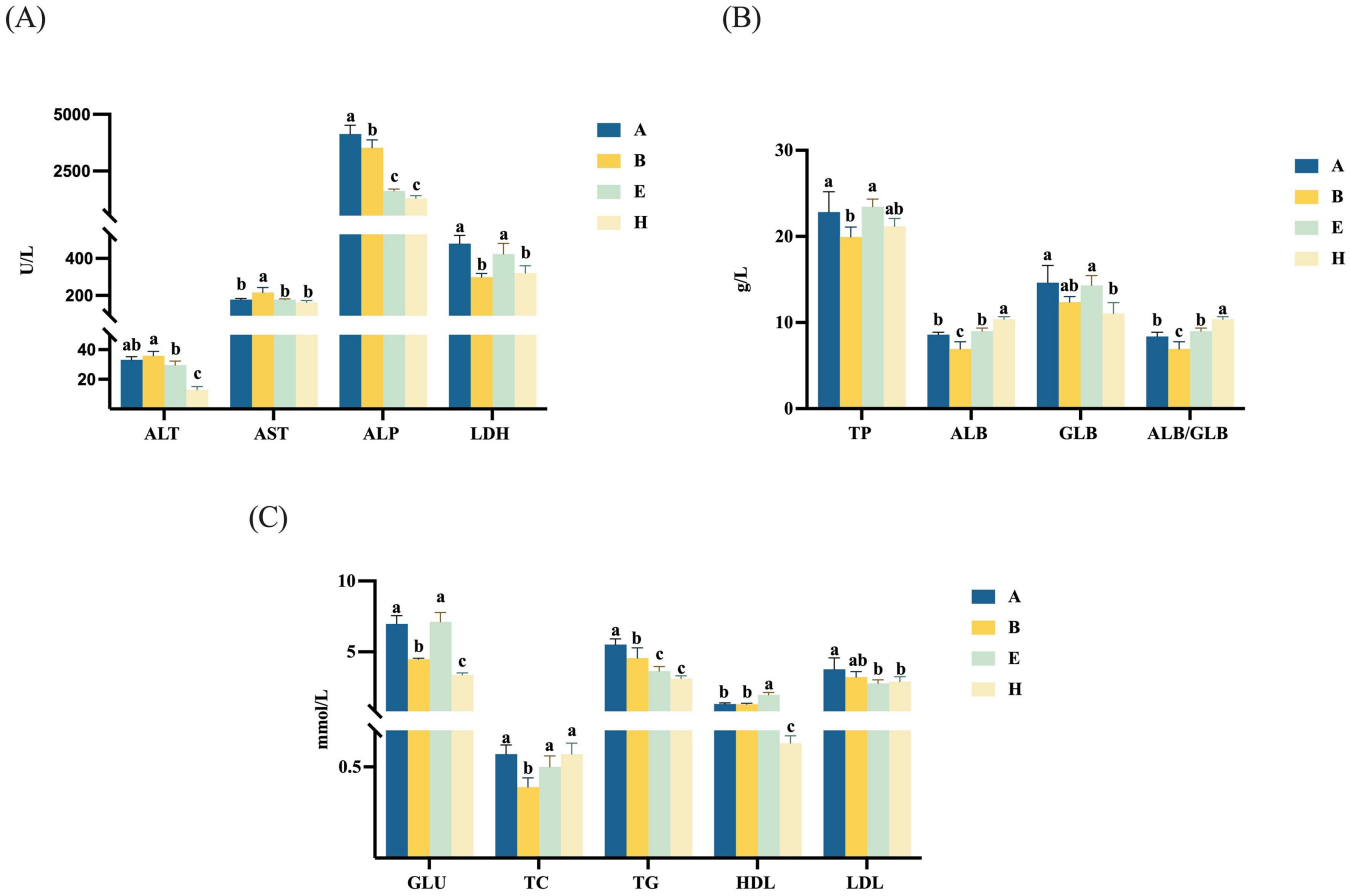
Effect of CANCGA on serum biochemical parameters of broilers (*n* = 5) **(A–C)**. A: basal diet; B: low-dose mycotoxin group; E: Basal diet + 0.4 g/kg CANCGA; H: Low-dose mycotoxin group + 0.4 g/kg CANCGA. ^a–c^ The values with different lowercase letters on each bar indicate significant difference (*p* < 0.05), whereas the values with the same lowercase letters on each bar indicate insignificant difference (*p* > 0.05).

Figure 1 indicated that serum ALP, LDH, TP, ALB, GLU, TC and TG levels in low-dose mycotoxin group were significantly decreased (*p* < 0.05), while AST level was significantly increased (*p* < 0.05), compared with the control group. However, the addition of 0.4 g/kg CANCGA to the basal diet could decrease serum ALP, TG and LDL levels (*p* < 0.05), while increase HDL level (*p* < 0.05). Compared with the low-dose mycotoxin group, the addition of 0.4 g/kg CANCGA could decrease serum ALT, AST, ALP, GLU, TG and HDL levels (*p* < 0.05), while increase ALB, TC levels and ALB/GLB (*p* < 0.05). It was inferred that CANCGA addition was able to reduce tissue and organ damage and lipid metabolism disorders of broilers induced by multi-mycotoxins.

### 3.5 Analysis of serum differential metabolites

**Figure 2:**
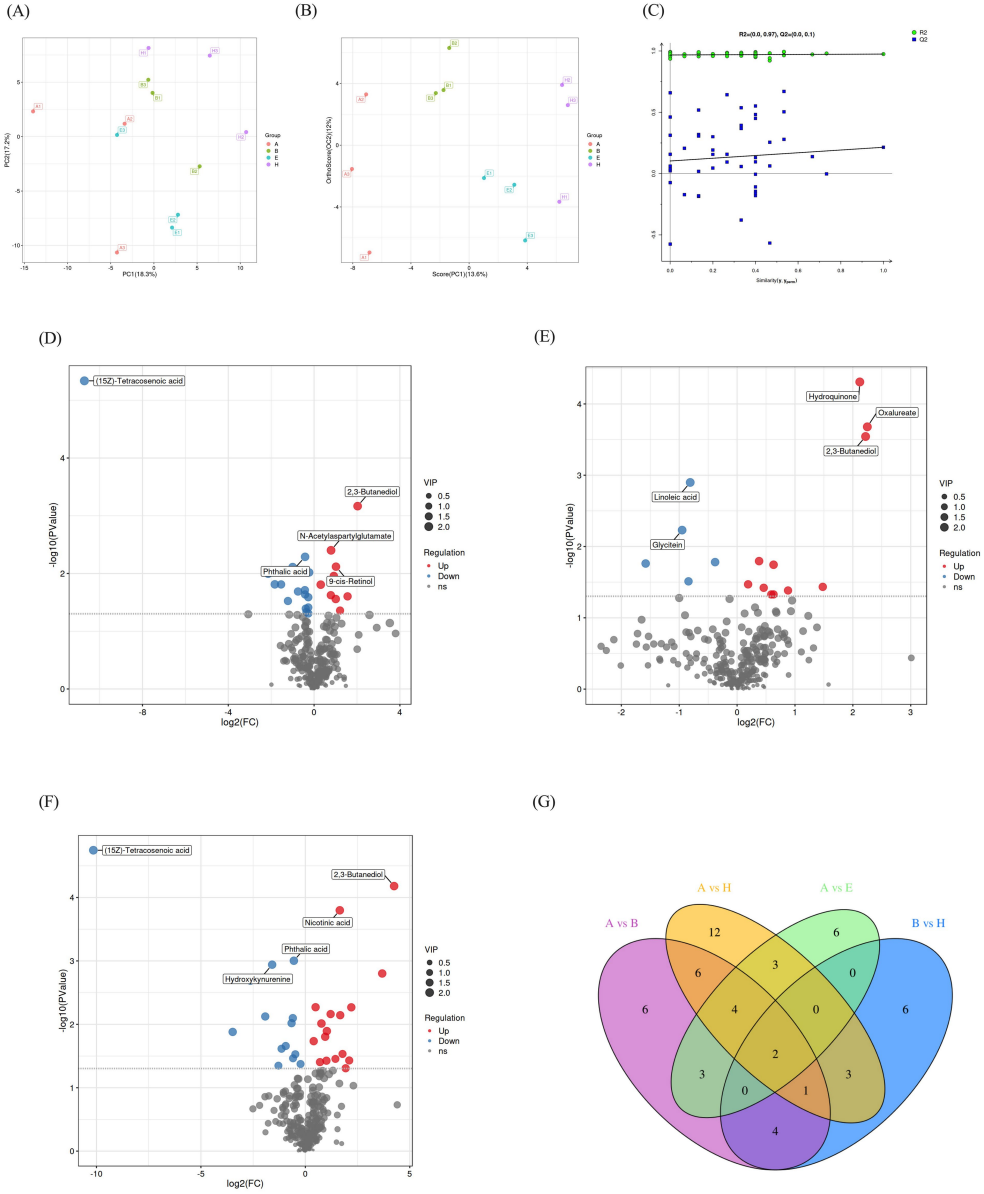
Comprehensive metabolic profiling and comparison of serum metabolites among different groups. Principal component analysis of serum metabolites **(A)**. OPLS-DA model analysis for each group **(B,C)**. Volcanic map of differences in metabolites between two groups. Differential metabolites in the comparisons of group B vs. group A, group H vs. group B, group H vs. group A, respectively **(D–F)**. Venn map of differential metabolites in group A vs. group B, group A vs. group H, group A vs. group E, group B vs. group H **(G)**.

In order to study the mechanism of CANCGA for alleviating multi-mycotoxin toxicity, nutrient metabolism of broilers was analyzed by UPLC-MS spectrometry (Figures 2A,B). In Figure 2C, the horizontal coordinates indicated the similarity between the real grouping of the samples and the 100 random groupings, the vertical coordinates indicated the model evaluation parameters, Q2 and R2 points in the upper right corner indicated the model evaluation parameters of the real grouping. If both Q2 fell below R2, it means that the results are reliable. The results of PCA and OPLS-DA in Figure 2 revealed notable differences of metabolites in broiler serum. In the comparisons of group B vs. group A, group H vs. group B, group H vs. group A, group E vs. group A, about 26, 16, 31 and 18 differential metabolites were identified, respectively (Table 5). Nine metabolites were significantly up-regulated, and seventeen metabolites were down-regulated in the comparison of group B vs. group A (*p* < 0.05). Seventeen metabolites were significantly up-regulated, and fourteen metabolites were down-regulated in the comparison of group H vs. group A (*p* < 0.05). Six metabolites were significantly up-regulated, and twelve metabolites were down-regulated in the comparison of group E vs. group A (*p* < 0.05). Eleven metabolites were significantly up-regulated, and five metabolites were down-regulated in the comparison of group H vs. group B (*p* < 0.05).

**Table 5:** The expression levels of differential metabolites in different groups.

Differential metabolites name	A^1^	B^2^	E^3^	H^4^	B vs. A	H vs. B	H vs. A	E vs. A
Dimethylglycine	8.44E+07	1.14E+08	1.01E+08	9.99E+07	Down	—	—	Down
5-Hydroxypentanoic acid	1.88E+07	2.26E+07	2.53E+07	2.42E+07	Down	—	—	—
L-Glutamic gamma-semialdehyde	1.58E+07	2.07E+07	2.30E+07	2.38E+07	Up	—	Up	—
Creatine	1.42E+08	6.15E+07	1.33E+08	4.45E+07	Down	—	Down	Down
2-Keto-glutaramic acid	2.08E+07	2.79E+07	2.79E+07	3.15E+07	Down	—	Down	Down
Phthalic acid	5.79E+07	7.73E+07	7.55E+07	8.45E+07	Up	Up	Up	Up
2,3-Butanediol	1.11E+08	2.72E+07	5.55E+07	5.84E+06	Down	—	—	Down
Cyromazine	6.57E+06	7.69E+06	8.68E+06	8.26E+06	Down	—	—	—
3-Indoleacrylate	1.24E+07	1.59E+07	1.60E+07	1.18E+07	Up	—	—	—
8-Amino-7-oxononanoate	2.03E+07	1.07E+07	2.50E+07	2.13E+07	Down	Down	Down	—
Selenocysteine	1.14E+07	3.30E+07	1.66E+07	4.30E+07	Down	—	Down	Down
Methoxamine	1.41E+08	1.69E+08	1.73E+08	1.64E+08	Down	—	Down	—
gamma-Glutamyl-beta-aminopropiononitrile	3.44E+06	1.51E+07	4.65E+06	2.13E+07	Down	Up	—	—
(−)-Jasmonic acid	6.37E+06	1.27E+07	6.84E+06	8.47E+06	Down	Down	Down	Down
Hydroxykynurenine	2.09E+07	3.53E+07	6.95E+07	6.31E+07	Up	Down	—	—
3-Ketosphingosine	4.48E+07	1.51E+07	1.34E+08	4.54E+07	Up	Down	—	—
Glycitein	2.17E+09	1.26E+09	2.78E+09	2.43E+09	Up	—	Up	—
9-cis-Retinol	1.70E+08	8.34E+07	1.16E+08	7.33E+07	Down	—	—	—
Aldosterone	1.69E+07	2.81E+07	3.16E+07	3.23E+07	Down	—	—	—
Antibiotic JI-20A	9.42E+07	2.19E+08	1.14E+08	1.79E+08	Down	—	Down	—
(R)-3-Hydroxybutyric acid	2.17E+07	7.70E+07	1.68E+07	2.42E+08	Down	—	—	—
Anserine	3.81E+06	4.59E+06	4.79E+06	2.50E+06	Up	—	Up	—
2-Methoxyestradiol	8.08E+06	6.53E+06	9.05E+06	6.13E+06	Up	—	—	Up
N-Acetylaspartylglutamate	6.89E+06	3.99E+06	4.76E+06	4.92E+06	Up	—	—	—
13-L-Hydroperoxylinoleic acid	1.87E+07	9.29E+06	1.69E+07	1.51E+07	DOWN	—	Down	Down
(15Z)-Tetracosenoic acid	2.32E+04	3.87E+07	3.06E+07	2.63E+07	DOWN	Up	Up	—
Hydroquinone	1.26E+08	1.44E+08	1.87E+08	3.30E+07	—	Up	—	—
L-Homoserine	1.12E+08	1.03E+08	1.65E+08	7.48E+07	—	Up	—	—
Oxalureate	4.16E+07	4.31E+07	2.42E+07	9.04E+06	—	Up	Up	—
Acetaminophen	1.70E+07	1.62E+07	1.85E+07	1.05E+07	—	Up	—	—
L-Kynurenine	5.86E+06	5.92E+06	9.90E+06	3.81E+06	—	Down	—	—
Linoleic acid	4.55E+07	3.08E+07	6.97E+07	5.41E+07	—	Up	—	—
beta-Carotene	4.53E+07	9.04E+07	3.63E+07	3.23E+07	—	Up	—	—
L-Methionine	2.91E+07	3.03E+07	3.18E+07	2.32E+07	—	Up	Up	—
N-Acetyl-D-tryptophan	1.03E+06	1.17E+06	7.04E+05	6.35E+05	—	—	Down	—
Dihydrouracil	8.58E+06	1.47E+07	1.09E+07	1.64E+07	—	—	Up	—
Nicotinic acid	9.60E+08	8.00E+08	5.88E+08	3.06E+08	—	—	Down	—
Anabasine	1.20E+07	1.45E+07	1.48E+07	1.66E+07	—	—	Down	Down
Gabapentin	3.57E+07	4.74E+07	5.13E+07	5.65E+07	—	—	Up	—
N-Acetylornithine	6.70E+07	5.48E+07	7.80E+07	4.77E+07	—	—	Up	—
Mannitol	4.28E+08	1.77E+08	2.18E+08	1.57E+08	—	—	Up	—
Se-Methylselenocysteine	9.46E+07	1.58E+07	3.15E+07	7.36E+06	—	—	Up	—
D-Octopine	3.05E+07	1.72E+07	3.90E+07	8.89E+06	—	—	Down	—
Alprenolol	6.20E+07	1.13E+08	8.31E+07	1.37E+08	—	—	Down	Down
Protoporphyrinogen IX	1.51E+09	3.38E+09	2.73E+09	3.69E+09	—	—	Up	—
Gallic acid	2.41E+07	1.51E+07	1.34E+07	1.42E+07	—	—	Up	—
N-Acetylleucine	2.97E+07	2.02E+07	3.90E+07	1.47E+07	—	—	Up	Up
Guanidinosuccinic acid	5.03E+08	4.34E+07	6.51E+07	1.18E+08	—	—	Up	—
N-Acetylanthranilate	3.90E+06	2.55E+06	2.32E+06	2.02E+06	—	—	Up	—
N-Acetyl-L-phenylalanine	8.37E+06	5.70E+06	7.44E+06	4.11E+06	—	—	—	Down
Indole	3.79E+07	4.41E+07	4.78E+07	4.50E+07	—	—	—	Down
Aminomalonic acid	1.28E+07	1.96E+07	8.42E+07	2.50E+07	—	—	—	Down
Guanosine	9.26E+06	1.95E+07	2.51E+07	1.92E+07	—	—	—	Up
Resveratrol	2.21E+07	2.42E+07	6.47E+06	1.37E+07	—	—	—	Up
Dehydroepiandrosterone	1.74E+07	1.15E+07	9.07E+06	1.47E+07	—	—	—	Up
Alpha-dimorphecolic acid	1.18E+07	7.09E+06	3.28E+06	2.05E+07	—	—	—	Down

1 A: basal diet.

2 B: low-dose mycotoxin group.

3 E: basal diet + 0.4 g/kg CANCGA.

4 H: low-dose mycotoxin group + 0.4 g/kg CANCGA.

The “E” in the table denotes scientific notation, where it represents 10 raised to the negative power of the given date; for example, “8.44E + 07” means 8.44 × 10−7.

The distribution and alteration of distinct metabolites between the experimental groups and the control group were illustrated in Figure 2D–F. Additionally, the top five metabolites exhibiting statistically significant differences among the groups were highlighted in the volcano plot. Compared to group A, (15Z)-tetracosenoic acid and phthalic acid were downregulated, while 2,3-butanediol, N-acetylaspartylglutamate and 9-cis-retinol were upregulated in group B (Figure 2D). Compared to group B, linoleic acid and glycitein were downregulated, while hydroquinone, oxalureate and 2,3-butanediol were upregulated in group H (Figure 2E). Furthermore, compared to group A, (15Z)-tetracosenoic acid, phthalic acid and hydroxykynurenine were downregulated, while 2,3-butanediol and nicotinic acid were upregulated in group H (Figure 2F). The differential metabolites in each group were analyzed using Venn plots (Figure 2G). In four comparisons such as group B vs. group A, group H vs. group B, group H vs. group A, group E vs. group A, two common differential metabolites (2,3-butanediol and hydroxykynurenine) were identified. Additionally, seven common metabolites (dimethylglycine, 2,3-butanediol, selenocysteine, jasmonic acid, hydroxykynurenine, 3-ketosphingosine and glycitein) were found in the comparisons of group B vs. group A, group H vs. group B.

### 3.6 KEGG enrichment analysis for serum differential metabolites

**Figure 3:**
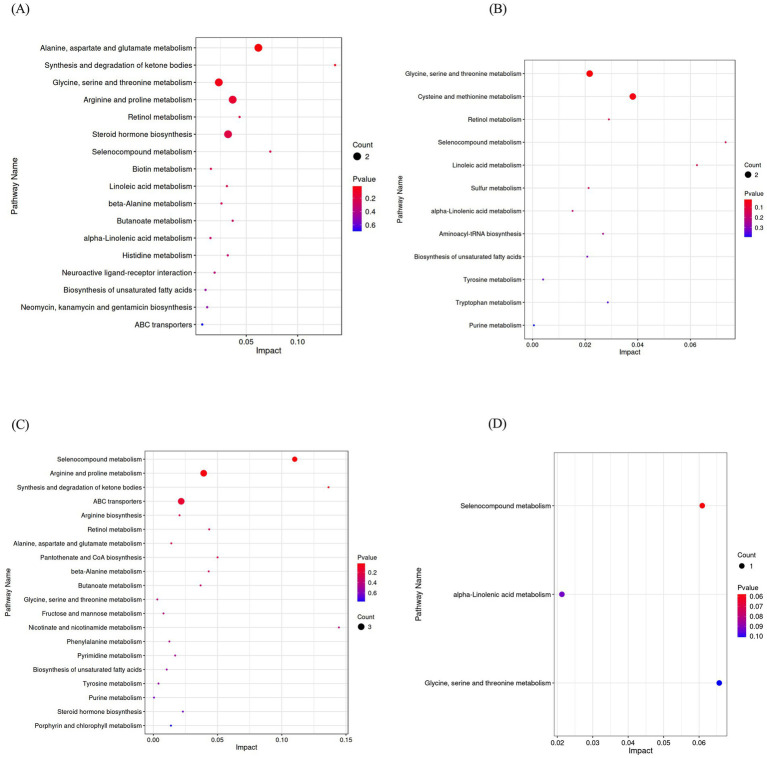
KEGG pathway enrichment of differential metabolites. Differential metabolites were enriched in KEGG pathways in group B vs. group A, group H vs. group B, group H vs. group A **(A–C)**; The differential metabolites were enriched in three pathways by KEGG analysis in group B vs. group A and group H vs. group B **(D)**.

To further elucidate the metabolic pathways associated with the differential metabolites in serum, KEGG pathway enrichment analysis was conducted for the following four comparisons: group B vs. group A, group H vs. group B, group H vs. group A, group E vs. group A (Figures 3A–D). Compared with group A, the differential metabolites in group B were primarily enriched in alanine, aspartate and glutamate metabolism pathway; glycine, serine and threonine metabolism pathway; arginine and proline metabolism pathway; steroid hormone biosynthesis pathway (Figure 3A). Compared with group B, the differential metabolites in group H were primarily enriched in the pathways related to glycine, serine and threonine metabolism as well as cysteine and methionine metabolism (Figure 3B). The differential metabolites observed in group H were predominantly associated with selenocompound metabolism, arginine and proline metabolism as well as ABC transporters, compared with group A (Figure 3C).

Further KEGG enrichment analysis was conducted for the common differential metabolites in the comparisons of group B vs. group A, group H vs. group B, revealing the significant associations with three pathways such as selenocompound metabolism, alpha-linolenic acid metabolism as well as glycine, serine and threonine metabolism (Figure 3D). These findings indicated that mycotoxins impacted amino acid synthesis and metabolism as well as steroid hormone biosynthesis pathways to induce organism damage. However, supplementation with CANCGA could mitigate mycotoxin-induced damage by modulating amino acid synthesis and metabolism pathways along with selenium complex metabolism and *α*-linolenic acid metabolism.

### 3.7 Correlation between serum differential metabolites and growth performance, serum biochemical parameters or toxin residues in excreta

**Figure 4:**
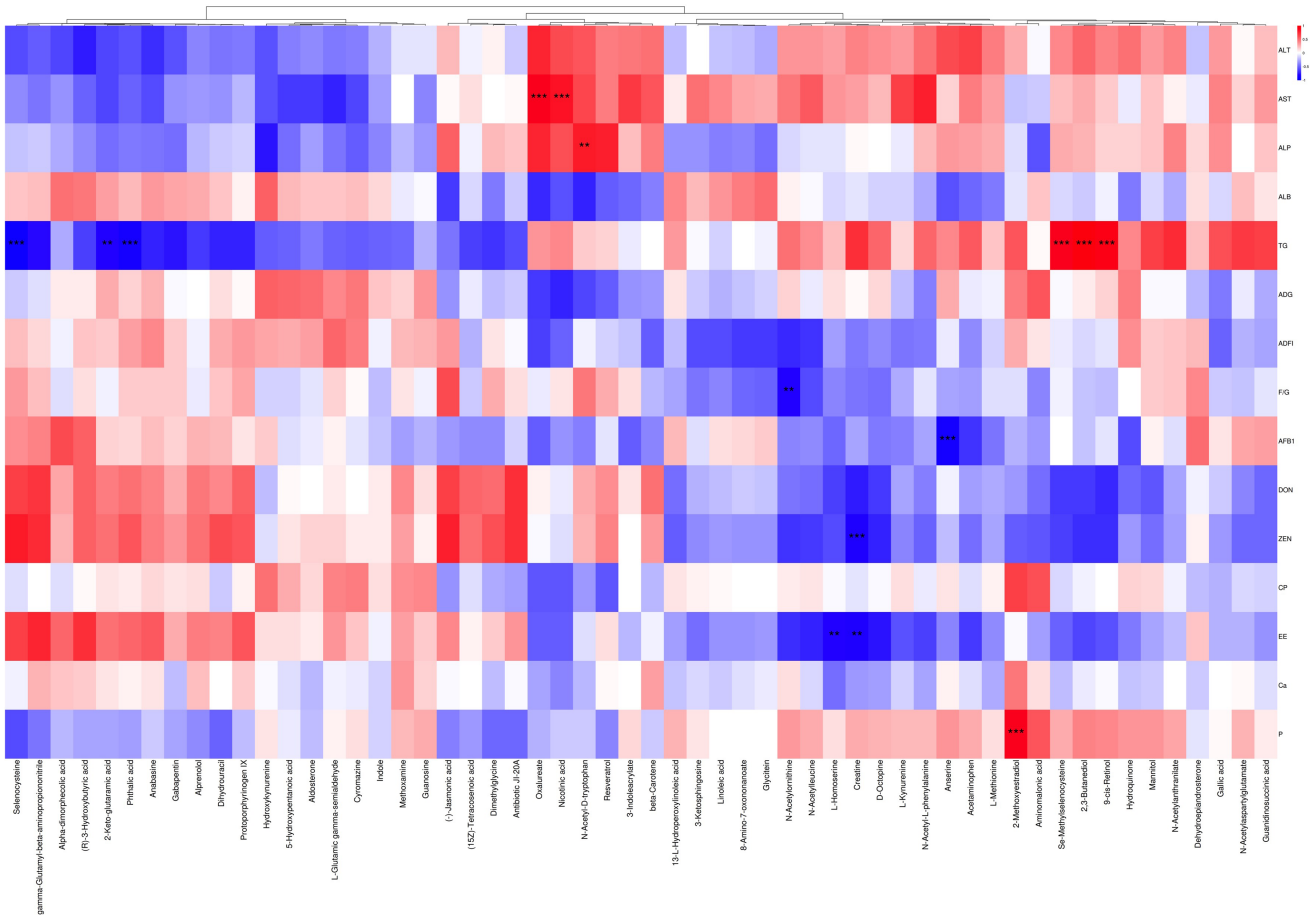
Correlation analysis between serum metabolites and other parameters. Red indicates positive correlation, blue indicates negative correlation; * represents *p* < 0.05, * * represents *p* < 0.01, and * * * represents *p* < 0.001. ALT: alanine aminotransferase; AST: aspartate aminotransferase; ALP: alkaline phosphatase; ALB: albumin; TG: triglycerides; ADG: average daily gain; ADFI: average daily feed intake; F/G: daily feed intake/daily gain; AFB_1_: aflatoxin B_1_ residue in excreta; DON: deoxynivalenol residue in excreta; ZEN: zearalenone residue in excreta; CP: crude protein metabolic rate; EE: ether extract metabolic rate; CA: calcium metabolic rate; P: phosphorus metabolic rate.

The relationships between differential metabolites in serum and serum biochemical indices, ADFI, ADG, nutrient metabolic rates or residual levels of mycotoxins in excreta were illustrated in Figure 4. Serum metabolites including 2,3-butanediol, 9-cis-retinol, and Se-methylselenocysteine were significantly positively correlated with serum TG level (*p* < 0.05). Conversely, selenocysteine, phthalic acid, and 2-keto-glutaramic acid were significantly negatively correlated with serum TG level (*p* < 0.05). Serum ALP level was positively correlated with the N-acetyl-D-tryptophan (*p* < 0.05). Serum AST level was significantly positively correlated with oxalureate and nicotinic acid (*p* < 0.05). Serum N-acetylornithine level was significantly negatively correlated with F/G (*p* < 0.05). Serum anserine level was significantly negatively correlated with AFB_1_ residue in excreta, while creatine was significantly negatively correlated with ZEN residue in excreta. Both serum creatine and L-homoserine levels showed significant negative correlations with EE metabolic rate, whereas P metabolic rate had a significant positive correlation with serum 2-methoxyestradiol level. The above results indicated that serum differential metabolites had close correlation with broiler growth performance, serum biochemical parameters or toxin residues in excreta.

## 4 Discussion

About 70% of contaminated feed ingredients contain more than one mycotoxin, posing a serious threat to animal health (1). Previous studies showed the potential of microbial products and plant extracts for this purpose (40). Combining plant extracts with microorganisms may enhance mycotoxin-degradation efficiency because plant extracts contain active compounds capable of binding toxin molecules or promoting their breakdown, thereby mitigating cellular and tissue damage from mycotoxins (41). The research in our laboratory demonstrated that *Aspergillus niger* solid cultures could degrade AFB_1_ and ZEN effectively. Another research showed that GA promoted cell proliferation enhanced intestinal barrier function, improved nutrient transport and absorption, and reduced DON-induced damage in piglet intestinal epithelial cells (38). Therefore, the combination of *Aspergillus niger* solid cultures with GA may be able to alleviate AFB_1_, ZEN and DON toxicity. This study proved the effectiveness of this combination in alleviating multi-mycotoxins toxicity for broilers. The reason may be due to the ability of *Aspergillus niger* cultures to degrade AFB_1_ and ZEN, as well as the GA favoring nutrient transport and absorption in the intestine and alleviating the impairment of nutrient transport by DON.

The consumption of mycotoxin-contaminated feed reduces growth performance and increases pathological traits in broilers (42, 43). High mortality and low ADG of broilers induced by multi-mycotoxins in this study correspond to the above reports. The reasons are that the mycotoxins often cause tissue and organ damage, impair nutrient digestion and utilization, and weaken immune function (44, 45). However, EE metabolic rate was increased with dietary mycotoxin levels increasing, likely due to ZEN similar to endogenous estrogen promoting fat metabolism (46). 0.04 and 0.06% CANCGA supplementation improved growth performance and nutrient metabolisms as well as reduced mortality and mycotoxin levels in feces for broilers exposed to mycotoxins, owing to mycotoxin degradation and detoxification of CANCGA. No significant differences in broiler growth performance were observed with higher or lower CANCGA additions, indicating that the lower cost CANCGA additions were also effective in alleviating the toxic impacts of multi-mycotoxins in production practice.

As mycotoxins are primarily metabolized in the liver, it is the main target for their toxic effects (47). Exposure to mycotoxin-contaminated diets elevated serum AST and ALT levels, leading to hepatic dysfunction, consistent with prior findings (48), and further supporting the link between organ damage and low broiler productivity due to mycotoxins (49). CANCGA addition significantly increased serum TP and ALB levels, while reduced serum AST, ALT and ALP levels, demonstrating the efficacy of CANCGA in mitigating tissue and organ damages induced by multi-mycotoxins, mainly due to the hepatoprotective activity of GA (50) and mycotoxin-degradation ability of *Aspergillus niger* culture.

To further investigate the impact of mycotoxin exposure and CANCGA supplementation on nutrient metabolisms in broilers, serum metabolomics was conducted in groups A, B, E, and H. The results demonstrated that mycotoxin exposure induced significant alterations in serum metabolites. Specifically, the upregulation of 2,3-butanediol and 9-cis-retinol indicated that mycotoxins disrupt intracellular energy and retinol metabolism, thereby exacerbating oxidative stress and inflammation (51, 52). Compared with the basal diet group, the downregulation of (15Z)-tetracosenoic acid and phthalic acid in the low mycotoxin group reflected the inhibition of normal fatty acid metabolism by mycotoxins, which promoted hepatic lipid accumulation (53) and aggravated liver injury. However, supplementation with CANCGA helped to restore these key metabolites to normal levels. The observed changes in antioxidant metabolites (hydroquinone and glycitein) (54) and lipid metabolites (linoleic acid) (55) suggested that the CANCGA mitigates mycotoxin-induced damage by modulating fatty acid metabolism and enhancing antioxidant defenses, ultimately contributing to the restoration of metabolic balance.

The KEGG pathway enrichment analysis revealed significant metabolic disturbances caused by multi-mycotoxins. These mycotoxins predominantly disrupted normal ketone body metabolism and impaired energy-metabolism stability by interfering with alanine, aspartate, and glutamate metabolisms as well as the synthesis and degradation of ketone bodies. This disruption led to inflammation and lipid metabolism disorders, exacerbating liver damage (56). The enrichments of glycine, serine and threonine metabolisms may indicate the organism’s response to oxidative stress and cellular damage caused by mycotoxins, potentially through the enhancement of antioxidant defense mechanisms. Following the addition of CANCGA, pathway enrichment analysis suggested a partial alleviation of the metabolic disturbances induced by mycotoxins.

This study showed that there were significant increases in metabolites related to glycine, serine and threonine metabolisms as well as cysteine and methionine metabolisms by CANCGA addition. Glycine, serine and threonine are critical amino acids involved in one-carbon metabolism, which is essential for DNA synthesis, repair and cell proliferation (57). Moreover, glycine exhibits detoxifying and hepatoprotective properties, while serine and cysteine are integral to the synthesis of glutathione (GSH), a principal antioxidant in the body that effectively neutralizes free radicals and safeguards cells from oxidative damage (58). Methionine is involved in methylation reactions, which are crucial for maintaining cellular function and stability (59). Consequently, CANCGA addition not only directly degrades mycotoxins but also facilitates the restoration of metabolic pathways, thereby enhancing the synthesis of antioxidants such as GSH and bolstering detoxification processes. It stimulated one-carbon metabolism, promoting cellular repair and regeneration, and mitigating the metabolic disturbances and hepatic damage induced by mycotoxins. Simultaneously, the enrichment of lipid metabolism-related pathways such as alpha-linolenic acid metabolism and unsaturated fatty acid biosynthesis by CANCGA addition indicated a reduction in lipid oxidation, leading to a more balanced fatty acid metabolism, thereby mitigating the impact of mycotoxins on lipid metabolism (60).

Furthermore, the enrichment of selenocompound metabolism by CANCGA addition was intricately linked to the body’s antioxidant capacity, immune regulation and anti-inflammatory responses (61). It is inferred that CANCGA modulated selenium metabolism, enhancing the synthesis and activity of antioxidant enzymes, reducing toxin-induced inflammation, and strengthening the body’s defense against oxidative stress caused by mycotoxins. In conclusion, the mycotoxin group exhibited significant disruptions in amino acid metabolism, lipid metabolism and antioxidant pathways, which were closely associated with oxidative stress, inflammation and hepatocellular damage caused by mycotoxins. After the addition of CANCGA, the disturbed metabolic pathways are restored to the normal levels, especially in anti-oxidative capacity and lipid metabolism regulation.

The differential metabolites common to the four groups were 2,3-butanediol and hydroxylurine. The production of 2,3-butanediol by intestinal microbes (62) suggested that the intestinal microbiome underwent adaptive changes to counteract mycotoxin-induced stress. CANCGA supplementation facilitated adjustments in the gut microflora without altering the protective role of 2,3-butanediol. Hydroxykynurenine, an intermediate in tryptophan metabolism (63), highlighted the tryptophan pathway’s role in sustaining antioxidant capacity and immune function regulated with or without CANCGA intervention.

The level of serum TG was found to be correlated with selenocysteine and methyl selenocysteine, both of which played a role in the selenium metabolism pathway identified through KEGG enrichment analysis. Previous study demonstrated that selenocysteine could reduce TG levels (64). This study showed that CANCGA supplementation increased the level of selenocysteine, resulting in low TG levels. Additionally, a significant positive correlation was observed between TG and 9-cis-retinol, suggesting a link between lipid metabolism and anti-oxidative function. It was reported that 9-cis-retinol regulated the expression of antioxidant genes, thereby enhancing cellular tolerance to oxidative stress (65). Moreover, lipid metabolism provided energy support for sustaining antioxidant and immune functions under mycotoxin stress. The strong positive correlation observed between AST and oxalureate suggested that mycotoxin-induced oxidative stress in the liver promoted purine metabolism, leading to high production of oxalureate (66). Furthermore, the positive association between AST and nicotinic acid implied that mycotoxin-induced stress might augment the demand for nicotinic acid to enhance NAD + synthesis, thereby bolstering cellular anti-oxidative capacity and facilitating liver and tissue resilience against oxidative stress (67).

The significant negative correlation between serum anserine and AFB_1_ residue in excreta suggested that the anti-oxidative properties of anserine might mitigate oxidative damage induced by AFB_1_, thereby reducing its presence in the body (68). Additionally, a significant negative correlation was observed between creatine level and ZEN residue in excreta. KEGG enrichment analysis revealed that creatine was involved in metabolic pathways related to glycine, serine, threonine, arginine and proline metabolisms, all of which occur primarily in the liver. Disruption of these pathways due to liver damage led to low creatine levels. CANCGA supplementation could restore the nutrient metabolic disorders induced by multi-mycotoxins.

## 5 Conclusion

The mortality was increased and growth performance was decreased when broilers were exposed to both low-dose and high-dose multi-mycotoxins. However, dietary CANCGA supplementation could decrease broiler mortality, improve growth performance and nutrient metabolic rates, and alleviate tissue and organ damages caused by mycotoxins. CANCGA ameliorated nutrient metabolic disorders induced by mycotoxins through modulation of pathways involved in glycine, serine and threonine metabolism, cysteine and methionine metabolism, selenium complex metabolism as well as linoleic acid metabolism. This study investigated the role of CANCGA and its mechanism in mitigating the negative effects of mycotoxins on broilers, assessed its value and effectiveness as a feed additive in practical production, and provided an effective mycotoxin mitigation strategy.

## Data Availability

The original contributions presented in the study are included in the article/supplementary material, further inquiries can be directed to the corresponding authors.

## References

[1] Arif M Iram A Bhutta MAK Naiel MAK Abd El-Hack ME Othman SI. The biodegradation role of *saccharomyces cerevisiae* against harmful effects of mycotoxin contaminated diets on broiler performance, immunity status, and carcass characteristics. Animals. (2020) 10:238. doi: 10.3390/ani10020238 32028628 PMC7070355

[2] Alshannaq A Yu J. Occurrence, toxicity, and analysis of major mycotoxins in food. Int J Environ Res Public Health. (2017) 14:632. doi: 10.3390/ijerph14060632, PMID: 28608841 PMC5486318

[3] Binder EM. Managing the risk of mycotoxins in modern feed production. Anim Feed Sci Tech. (2007) 133:149–66. doi: 10.1016/j.anifeedsci.2006.08.008

[4] Liu M Zhang L Mo Y Li J Yang J Wang J. Ferroptosis is involved in deoxynivalenol-induced intestinal damage in pigs. J Anim Sci Biotechnol. (2023) 14:29. doi: 10.1186/s40104-023-00841-4, PMID: 36922863 PMC10018831

[5] Wang J Qu J Liu S Xu Q Li X Zhu Y. Tannic acid ameliorates systemic glucose and lipid metabolic impairment induced by low-dose t-2 toxin exposure. J Agric Food Chem. (2023) 71:12574–86. doi: 10.1021/acs.jafc.3c02934, PMID: 37525894

[6] Juan C Covarelli L Beccari G Colasante V Mañes J. Simultaneous analysis of twenty-six mycotoxins in durum wheat grain from Italy. Food Control. (2016) 62:322–9. doi: 10.1016/j.foodcont.2015.10.032

[7] Svihus B Uhlen AK Harstad OM. Effect of starch granule structure, associated components and processing on nutritive value of cereal starch: a review. Anim Feed Sci Tech. (2005) 122:303–20. doi: 10.1016/j.anifeedsci.2005.02.025

[8] Gruber-Dorninger C Jenkins T Schatzmayr G. Global mycotoxin occurrence in feed: a ten-year survey. Toxins. (2019) 11:375. doi: 10.3390/toxins11070375, PMID: 31252650 PMC6669473

[9] Streit E Naehrer K Rodrigues I Schatzmayr G. Mycotoxin occurrence in feed and feed raw materials worldwide: long-term analysis with special focus on Europe and Asia. J Sci Food Agr. (2013) 93:2892–9. doi: 10.1002/jsfa.6225, PMID: 23670211

[10] Karsauliya K Yahavi C Pandey A Bhateria M Sonker AK Pandey H. Co-occurrence of mycotoxins: a review on bioanalytical methods for simultaneous analysis in human biological samples, mixture toxicity and risk assessment strategies. Toxicon. (2022) 218:25–39. doi: 10.1016/j.toxicon.2022.08.016, PMID: 36049662

[11] Lumsangkul C Chiang HI Lo NW Fan YK Ju JC. Developmental toxicity of mycotoxin *fumonisin b₁* in animal embryogenesis: an overview. Toxins. (2019) 11:114. doi: 10.3390/toxins11020114, PMID: 30781891 PMC6410136

[12] Lumsangkul C Tso KH Fan YK Chiang HI Ju JC. Mycotoxin fumonisin B (1) interferes sphingolipid metabolisms and neural tube closure during early embryogenesis in brown tsaiya ducks. Toxins. (2021) 13:743. doi: 10.3390/toxins13110743, PMID: 34822527 PMC8619080

[13] Yang C Song G Lim W. Effects of mycotoxin-contaminated feed on farm animals. J Hazard Mater. (2020) 389:122087. doi: 10.1016/j.jhazmat.2020.122087, PMID: 32004836

[14] Eshetu E Habtamu A Gebretensa A. An overview on major mycotoxin in animal: its public health implication, economic impact and control strategies. J Health Med Nur. (2016) 25:64–73. doi: 10.1186/s41043-016-0050-6

[15] Holanda DM Kim SW. Mycotoxin occurrence, toxicity, and detoxifying agents in pig production with an emphasis on deoxynivalenol. Toxins. (2021) 13:171. doi: 10.3390/toxins13020171, PMID: 33672250 PMC7927007

[16] Dou X Wu G Ding Z Xie J. Construction of a nanoscale metal-organic framework aptasensor for fluorescence ratiometric sensing of AFB1 in real samples. Food Chem. (2023) 416:135805. doi: 10.1016/j.foodchem.2023.135805, PMID: 36878118

[17] Liu H Xie R Huang W Yang Y Zhou M Lu B. Negative effects of aflatoxin B1 (AFB1) in the diet on growth performance, protein and lipid metabolism, and liver health of juvenile hybrid grouper (*Epinephelus fuscoguttatus*♀×*Epinephelus lanceolatus*♂). Aquaculture Rep. (2023) 33:101779. doi: 10.1016/j.aqrep.2023.101779, PMID: 40642424

[18] Martínez J Hernández-Rodríguez M Méndez-Albores A Téllez-Isaías G Mera Jiménez E Nicolás-Vázquez MI. Computational studies of aflatoxin B1 (AFB1): a review. Toxins. (2023) 15:135. doi: 10.3390/toxins15020135, PMID: 36828449 PMC9967988

[19] Hou YJ Zhu CC Xu YX Cui XS Kim NH Sun SC. Zearalenone exposure affects mouse oocyte meiotic maturation and granulosa cell proliferation. Environ Toxicol. (2015) 30:1226–33. doi: 10.1002/tox.21995, PMID: 24733567

[20] Ropejko K Twarużek M. Zearalenone and its metabolites-general overview, occurrence, and toxicity. Toxins. (2021) 13:35. doi: 10.3390/toxins13010035, PMID: 33418872 PMC7825134

[21] Tatay E Espín S García-Fernández AJ Ruiz MJ. Oxidative damage and disturbance of antioxidant capacity by zearalenone and its metabolites in human cells. Toxicol In Vitro. (2017) 45:334–9. doi: 10.1016/j.tiv.2017.04.026, PMID: 28477956

[22] Ghareeb K Awad WA Böhm J Zebeli Q. Impacts of the feed contaminant deoxynivalenol on the intestine of monogastric animals: poultry and swine. J Appl Toxicol. (2015) 35:327–37. doi: 10.1002/jat.3083, PMID: 25352520

[23] Shen YB Weaver AC Kim SW. Physiological effects of deoxynivalenol from naturally contaminated corn on cerebral tryptophan metabolism, behavioral response, gastrointestinal immune status and health in pigs following a pair-feeding model. Toxins. (2021) 13:393. doi: 10.3390/toxins13060393, PMID: 34070838 PMC8230096

[24] Vignal C Djouina M Pichavant M Caboche S Waxin C Beury D. Chronic ingestion of deoxynivalenol at human dietary levels impairs intestinal homeostasis and gut microbiota in mice. Arch Toxicol. (2018) 92:2327–38. doi: 10.1007/s00204-018-2228-6, PMID: 29804187

[25] Peng Z Chen L Nüssler AK Liu L Yang W. Current sights for mechanisms of deoxynivalenol-induced hepatotoxicity and prospective views for future scientific research: a mini review. J Appl Toxicol. (2017) 37:518–29. doi: 10.1002/jat.3428 27996102

[26] Wang X Chen X Cao L Zhu L Zhang Y Chu X. Mechanism of deoxynivalenol-induced neurotoxicity in weaned piglets is linked to lipid peroxidation, dampened neurotransmitter levels, and interference with calcium signaling. Ecotoxicol Environ Saf. (2020) 194:110382. doi: 10.1016/j.ecoenv.2020.110382, PMID: 32146195

[27] Chang J Wang T Wang P Yin Q Liu C Zhu Q. Compound probiotics alleviating aflatoxin B_1_ and zearalenone toxic effects on broiler production performance and gut microbiota. Ecotoxicol Environ Saf. (2020) 194:110420. doi: 10.1016/j.ecoenv.2020.110420, PMID: 32151861

[28] Ma R Zhang L Liu M Su YT Xie WM Zhang NY. Individual and combined occurrence of mycotoxins in feed ingredients and complete feeds in China. Toxins. (2018) 10:113. doi: 10.3390/toxins10030113, PMID: 29518909 PMC5869401

[29] Wu L Li J Li Y Li T He Q Tang Y. Aflatoxin B (1), zearalenone and deoxynivalenol in feed ingredients and complete feed from different province in China. J Anim Sci Biotechnol. (2016) 7:63–10. doi: 10.1186/s40104-016-0122-8, PMID: 27790372 PMC5075205

[30] Tsiouris V Tassis P Raj J Mantzios T Kiskinis K Vasiljević M. Investigation of a novel multicomponent mycotoxin detoxifying agent in amelioration of mycotoxicosis induced by aflatoxin-b1 and ochratoxina in broiler chicks. Toxins. (2021) 13:367. doi: 10.3390/toxins13060367, PMID: 34064255 PMC8224362

[31] Wang Q Zhang Y Zheng N Zhao S Li S Wang J. The biochemical and metabolic profiles of dairy cows with mycotoxins-contaminated diets. PeerJ. (2020) 8:e8742. doi: 10.7717/peerj.8742, PMID: 32257637 PMC7103205

[32] Wu X Guo L Huang G Tang W Zhao S Wang J. Effects of dietary natural mycotoxins exposure on performance, biochemical parameters and milk small molecule metabolic pathways of lactating cows. Agric Basel. (2022) 12:420. doi: 10.3390/agriculture12030420

[33] Čolović R Puvača N Cheli F Avantaggiato G Greco D Đuragić O. Decontamination of mycotoxin-contaminated feedstuffs and compound feed. Toxins. (2019) 11:617. doi: 10.3390/toxins11110617, PMID: 31731462 PMC6891401

[34] Xing F Wang L Liu X Selvaraj JN Wang Y Zhao Y. Aflatoxin B1 inhibition in *aspergillus flavus* by *Aspergillus niger* through down-regulating expression of major biosynthetic genes and AFB1 degradation by atoxigenic *A. flavus*. Int J Mol Sci. (2017) 256:1–10. doi: 10.1016/j.ijfoodmicro.2017.05.013 28578264

[35] Ji J Yu J Yang Y Yuan X Yang J Zhang Y. Exploration on the enhancement of detoxification ability of zearalenone and its degradation products of *aspergillus niger* fs10 under directional stress of zearalenone. Toxins. (2021) 13:720. doi: 10.3390/toxins13100720, PMID: 34679013 PMC8537726

[36] Wang Q Huang Y Li Y Zhang L Tang H Zhang J. Glycyrrhizic acid mitigates tripterygium-glycoside-tablet-induced acute liver injury via PKM2 regulated oxidative stress. Meta. (2022) 12:1128. doi: 10.3390/metabo12111128, PMID: 36422270 PMC9694034

[37] Yuan T Wang J Chen L Shan J Di L. Glycyrrhizic acid improving the liver protective effect by restoring the composition of *Lactobacillus*. J Funct Foods. (2019) 52:219–27. doi: 10.1016/j.jff.2018.11.001

[38] Xu X Yan G Chang J Wang P Yin Q Liu C. Comparative transcriptome analysis reveals the protective mechanism of glycyrrhinic acid for deoxynivalenol-induced inflammation and apoptosis in IPEC-J2 cells. Oxidative Med Cell Longev. (2020) 2020:5974157. doi: 10.1155/2020/5974157, PMID: 33163144 PMC7604610

[39] Huang W Chang J Wang P Liu C Yin Q Song A. Effect of compound probiotics and mycotoxin degradation enzymes on alleviating cytotoxicity of swine jejunal epithelial cells induced by aflatoxin b₁ and zearalenone. Toxins. (2019) 11:12. doi: 10.3390/toxins11010012, PMID: 30609651 PMC6356961

[40] Ndiaye S Zhang M Fall M Ayessou NM Zhang Q Li P. Current review of mycotoxin biodegradation and bioadsorption: microorganisms, mechanisms, and main important applications. Toxins. (2022) 14:729. doi: 10.3390/toxins14110729, PMID: 36355979 PMC9694041

[41] Arimboor R. Metabolites and degradation pathways of microbial detoxification of aflatoxins: a review. Mycotoxin Res. (2024) 40:71–83. doi: 10.1007/s12550-023-00515-0, PMID: 38151634

[42] Haque MA Wang Y Shen Z Li X Saleemi MK He C. Mycotoxin contamination and control strategy in human, domestic animal and poultry: a review. Microb Pathog. (2020) 142:104095. doi: 10.1016/j.micpath.2020.104095, PMID: 32097745

[43] Sun Y Huang K Long M Yang S Zhang Y. An update on immunotoxicity and mechanisms of action of six environmental mycotoxins. Food Chem Toxicol. (2022) 163:112895. doi: 10.1016/j.fct.2022.112895, PMID: 35219766

[44] Magnoli AP Poloni VL Cavaglieri L. Impact of mycotoxin contamination in the animal feed industry. Curr Opin Food Sci. (2019) 29:99–108. doi: 10.1016/j.cofs.2019.08.009

[45] Mohaghegh A Chamani M Shivazad M Sadeghi AA Afzali N. Effect of esterified glucomannan on broilers exposed to natural mycotoxin-contaminated diets. J Appl Anim Res. (2017) 45:285–91. doi: 10.1080/09712119.2016.1174122

[46] Torres Irizarry VC Jiang Y He Y Xu P. Hypothalamic estrogen signaling and adipose tissue metabolism in energy homeostasis. Front Endocrinol. (2022) 13:898139. doi: 10.3389/fendo.2022.898139, PMID: 35757435 PMC9218066

[47] Hasuda AL Person E Khoshal AK Bruel S Puel S Oswald IP. Deoxynivalenol induces apoptosis and inflammation in the liver: analysis using precision-cut liver slices. Food Chem Toxicol. (2022) 163:112930. doi: 10.1016/j.fct.2022.112930, PMID: 35314294

[48] Saminathan M Selamat J Abbasi Pirouz A Abdullah N Zulkifli I. Effects of nano-composite adsorbents on the growth performance, serum biochemistry, and organ weights of broilers fed with aflatoxin-contaminated feed. Toxins. (2018) 10:345. doi: 10.3390/toxins10090345, PMID: 30150553 PMC6162765

[49] Lee J Jessen K Beltran R Starkl V Schatzmayr G Borutova R. Mycotoxin-contaminated diets and deactivating compound in laying hens: 1. Effects on performance characteristics and relative organ weight. Poultry Sci. (2012) 91:2089–95. doi: 10.3382/ps.2012-02136, PMID: 22912441

[50] Yu JY Ha JY Kim KM Jung YS Jung JC Oh S. Anti-inflammatory activities of licorice extract and its active compounds, glycyrrhizic acid, liquiritin and liquiritigenin, in BV2 cells and mice liver. Molecules. (2015) 20:13041–54. doi: 10.3390/molecules200713041, PMID: 26205049 PMC6332102

[51] Tabbaa SM Guilak F Lemmerman LR Glembotski N D'Lima DD Wang T. Elevated lipid metabolites in stored clinical OCA media correlate with chondrocyte death. Am J Sports Med. (2024) 52:2119–28. doi: 10.1177/03635465241252653 38857056

[52] Wu G Bazer FW Davis TA Kim SW Li P Marc Rhoads J. Arginine metabolism and nutrition in growth, health and disease. Amino Acids. (2009) 37:153–68. doi: 10.1007/s00726-008-0210-y, PMID: 19030957 PMC2677116

[53] Bednarski T Mohsin R Jamey Y. Short-term effect of saturated and monounsaturated fatty acids on hepatic energy metabolism. Diabetes. (2019) 68:285–LB. doi: 10.2337/db19-285-LB, PMID: 39502451

[54] Biais B Krisa S Cluzet S Da Costa G Waffo-Teguo P Mérillon J-M. Antioxidant and cytoprotective activities of grapevine stilbenes. J Agric Food Chem. (2017) 65:4952–60. doi: 10.1021/acs.jafc.7b01254 28551990

[55] Huyan Z Pellegrini N Steegenga W Capuano E. Insights into gut microbiota metabolism of dietary lipids: the case of linoleic acid. Food Funct. (2022) 13:4513–26. doi: 10.1039/d1fo04254h, PMID: 35348564

[56] Zhou C Hu L Mu R Mei X Wu X Wang C. Compound green tea (CGT) regulates lipid metabolism in high-fat diet induced mice. RSC Adv. (2022) 12:24301–10. doi: 10.1039/d2ra02831j, PMID: 36128535 PMC9412714

[57] Pan S Fan M Liu Z Li X Wang H. Serine, glycine and one-carbon metabolism in cancer (review). Int J Oncol. (2021) 58:158–70. doi: 10.3892/ijo.2020.5158, PMID: 33491748 PMC7864012

[58] Lv XC Wu Q Cao YJ Lin YC Guo WL Rao PF. Ganoderic acid a from *ganoderma lucidum* protects against alcoholic liver injury through ameliorating the lipid metabolism and modulating the intestinal microbial composition. Food Funct. (2022) 13:5820–37. doi: 10.1039/d1fo03219d, PMID: 35543349

[59] Elango R. Methionine nutrition and metabolism: insights from animal studies to inform human nutrition. J Nutr. (2020) 150:2518s–23s. doi: 10.1093/jn/nxaa155, PMID: 33000159

[60] Yi S Mai T Fang Y Tian Q Zhao S. Repeated injection of xylazine causes liver injury through the PPAR signaling pathway in rats. J Biochem Mol Toxicol. (2025) 39:e70101. doi: 10.1002/jbt.70101, PMID: 39692361

[61] Saeedi M Soltani F Babalar M Wiesner-Reinhold M Baldermann S Mastinu A. Selenium enhances growth, phenolic compounds, antioxidant capacity in *brassica oleracea* var. Chem Biodivers. (2024) 22:e202401731. doi: 10.1002/cbdv.202401731 39373226

[62] Ujlaki G Kovács T Vida A Kókai E Rauch B Schwarcz S. Identification of bacterial metabolites modulating breast cancer cell proliferation and epithelial-mesenchymal transition. Molecules. (2023) 28:5898. doi: 10.3390/molecules28155898, PMID: 37570868 PMC10420980

[63] Knubel CP Insfran C Martinez FF Diaz Lujan C Fretes RE Theumer MG. 3-Hydroxykynurenine, a tryptophan metabolite generated during the infection, is active against trypanosoma cruzi. ACS Med Chem Lett. (2017) 8:757–61. doi: 10.1021/acsmedchemlett.7b00169, PMID: 28740612 PMC5512135

[64] Sun X Yue SZ Qiao YH Sun ZJ Wang C Li HF. Dietary supplementation with selenium-enriched earthworm powder improves antioxidative ability and immunity of laying hens. Poultry Sci. (2020) 99:5344–9. doi: 10.1016/j.psj.2020.07.030, PMID: 33142450 PMC7647737

[65] Elomda AM Saad MF Saeed AM Elsayed A Abass AO Safaa HM. Antioxidant and developmental capacity of retinol on the in vitro culture of rabbit embryos. Zygote. (2018) 26:326–32. doi: 10.1017/s0967199418000308, PMID: 30289099

[66] Rusz M Del Favero G El Abiead Y Gerner C Keppler BK Jakupec MA. Morpho-metabotyping the oxidative stress response. Sci Rep. (2021) 11:15471. doi: 10.1038/s41598-021-94585-8, PMID: 34326354 PMC8322264

[67] Romani M Hofer DC Katsyuba E Auwerx J. Niacin: an old lipid drug in a new NAD (+) dress. J Lipid Res. (2019) 60:741–6. doi: 10.1194/jlr.S092007, PMID: 30782960 PMC6446705

[68] Tamura Y Takenaka S Sugiyama S Nakayama R. Occurrence of anserine as an antioxidative dipeptide in a red alga, *porphyra yezoensis*. Biosci Biotechnol Biochem. (1998) 62:561–3. doi: 10.1271/bbb.62.561, PMID: 27315933

